# Experimental Demonstration of a Spectral Fingerprint for the Saddle and Inverted Conformations of Porphyrins on Copper

**DOI:** 10.1021/acs.jpclett.5c00498

**Published:** 2025-05-12

**Authors:** Eleanor S. Frampton, Ailish Gray, Michael Clarke, Matthew Edmondson, Jonathan Bradford, David A. Duncan, Alex Saywell

**Affiliations:** † MAX IV Laboratory, 226073 Lund University, 22100 Lund, Sweden; ‡ School of Physics and Astronomy, 6123 University of Nottingham, University Park, Nottingham NG7 2RD, United Kingdom; § School of Chemistry, University of Nottingham, University Park, Nottingham NG7 2RD, United Kingdom; ∥ Diamond Light Source, Harwell Science and Innovation Campus, Fermi Avenue, Didcot OX11 0DE, United Kingdom

## Abstract

Combined density functional theory (DFT) and X-ray standing wave (XSW) studies have previously provided evidence for the preferential adoption of an inverted conformation of 2H-TPP on Cu(111) in contrast to the saddle conformation usually favored by porphyrin molecules adsorbed on metals. We experimentally demonstrate, via X-ray photoelectron spectroscopy (XPS) analysis, that the binding energies of the aminic and iminic nitrogen species provide a spectral fingerprint for both inverted and saddle conformations, as predicted by DFT studies. Our complementary scanning tunneling microscopy (STM) characterization also reveals conversion from the saddle to inverted conformation at an elevated temperature for an analogous porphyrin species (Br_2_TPP).

## Graphical abstract

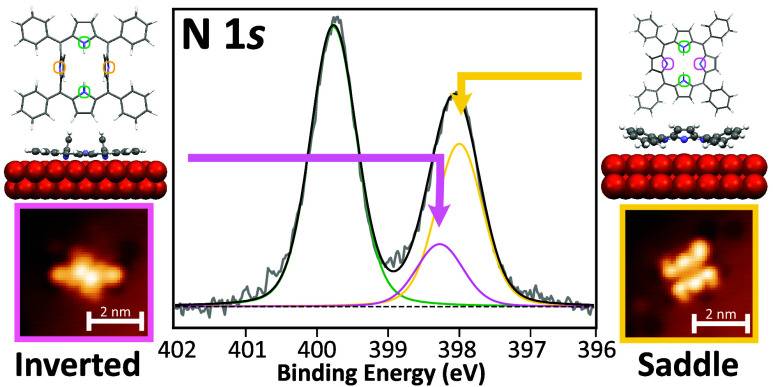

The on-surface chemistry of porphyrins and porpyrinoids is widely studied due to potential applications in organic semiconductors, heterogeneous catalysis, and molecular devices.^1–6^ They can undergo various on-surface reactions resulting in the formation of two-dimensional (2D) arrays and nanostructures with distinct properties.^7,8^ In particular, for applications in the field of molecular electronics, the interface between the porphyrin assembly and the supporting substrate can play a significant role: a weak molecule–substrate interaction is potentially a barrier to charge transport, whereas an overly strong interaction can alter the electronic properties and, therefore, the efficacy of the device.^9^ The capability to study these changes is key to enabling the design of molecule–substrate systems relevant for device applications.

Porphyrin molecules are well-known to adsorb at metal surfaces in a saddle-shaped conformation.^10–12^ In this conformation, two of the four pyrrolic rings are tilted toward the surface and two are tilted away, giving rise to the structures shown in Figure 1a. This deformation of the porphyrin upon adsorption is a result of conformational flexibility imbued by the rotations possible around multiple bonds, as shown in Figure S1. A series of studies over the last 10 years have shown that, when adsorbed on Cu surfaces, free-base tetraphenyl porphyrin (2H-TPP, structure shown in Figure S1) adopts an alternative to the saddle-shaped structure, a so-called “inverted” conformation.^13–15^ Iminic pyrrole rings display a near 90° rotation out of the plane of the molecule, breaking the aromaticity of the macrocycle, shown in Figure 1a and Figure S2. Consequently, the iminic nitrogen atoms within pyrrolic rings point directly toward the surface, facilitating a strong interaction with the substrate. Although the bond rotation required to convert between saddle and inverted conformations may have a significant associated energy barrier for gas phase systems,^16^ density functional theory (DFT) calculations indicate that, on Cu(111), the inverted structure is energetically favorable.^13^ This adsorption conformation was proposed in a number of studies supported by scanning tunneling microscopy (STM), DFT, and X-ray photoelectron spectroscopy (XPS) and later confirmed using X-ray standing wave (XSW).^17–19^

**Figure 1:**
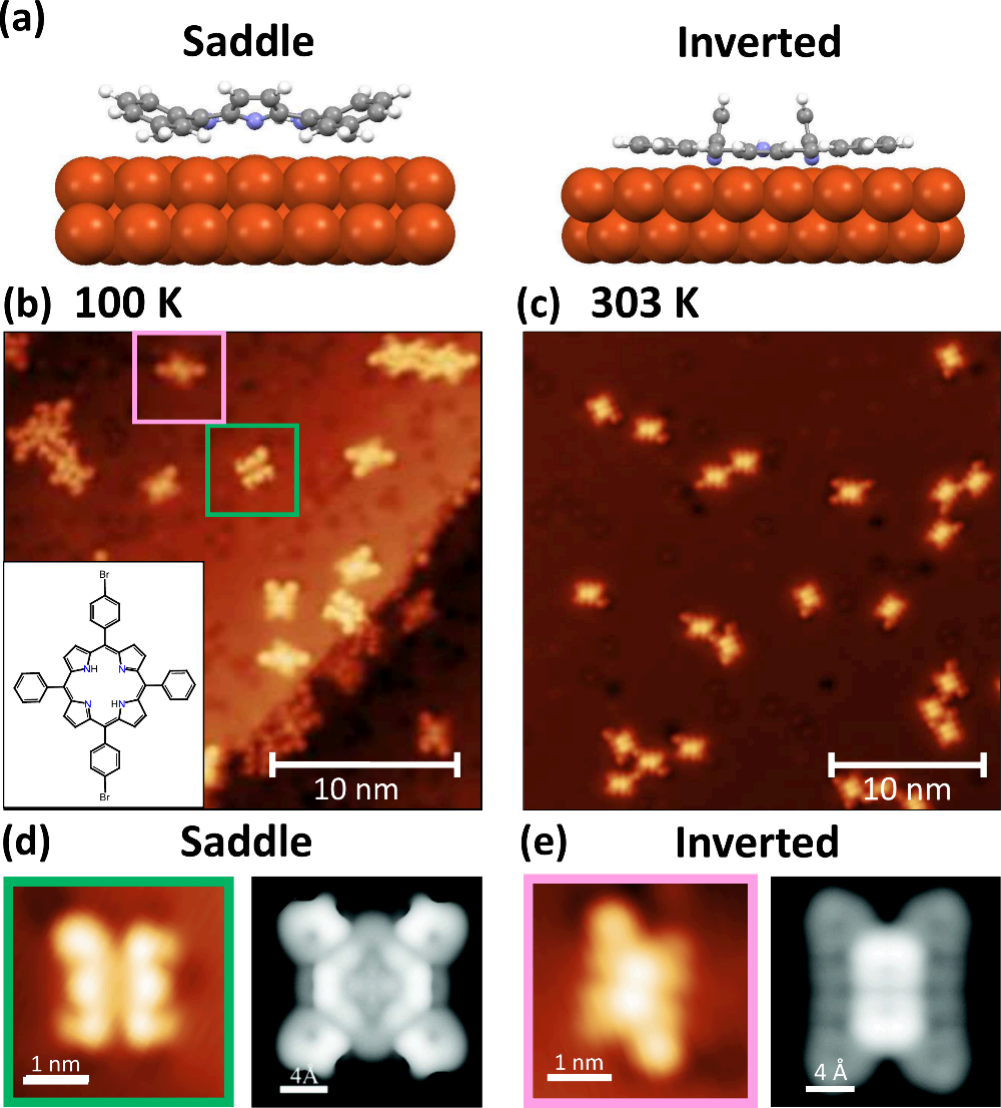
(a) Adsorption geometry of the DFT-derived saddle and inverted conformations of 2H-TPP on Cu(111). Panel a was reproduced with permission from ref 13. Copyright 2017 Royal Society of Chemistry. (b) STM topograph of as-deposited Br_2_TPP on Cu(111) with type 1 (pink) and type 2 (green) molecular contrast indicated. Bias = −1.6 V and set point = 40 pA. (Inset) Chemical structure of Br_2_TPP. (c) STM topograph of Br_2_TPP on Cu(111) following heating to 303 K. Bias = −1.6 V and set point = 350 pA. (d and e) Details of the molecular contrasts highlighted in panel b alongside DFT-calculated simulated STM images of each conformation. Panels d and e were reproduced with permission from ref 13. Copyright 2017 Royal Society of Chemistry.

This “inverted” model was introduced by Albrecht et al. and Moreno-Lopez et al. to explain their STM and non-contact atomic force microscopy (nc-AFM) results.^14,15^ In a subsequent paper by Lepper et al., DFT calculations demonstrated that the adsorption height determined by a prior XSW study could only be rationalized by this inverted model.^13^ Furthermore, these DFT calculations predicted that the distortion of the macrocycle would result in the iminic N atom being displaced from an off-atop site into a surface bridge site [confirmed by further normal incidence X-ray standing wave (NIXSW) triangulation measurements by Ryan et al.^17^]. However, in the work by Lepper et al., a consequence of the proposed inverted model was a DFT-predicted binding energy shift in the N 1s spectra between the aminic (N–H) and iminic (N=) nitrogen species. Calculations predicted that a saddle-shaped adsorption on Cu(111) should result in a binding energy (BE) shift of ∼2 eV but that the inverted model should have a far smaller BE shift of ∼1.5 eV. In combination with this binding energy shift, a significant difference in contrast was predicted in simulated STM images of saddle and inverted conformations of 2H-TPP on Cu(111). To date, this binding energy shift between these conformations has not been observed experimentally. Indeed, it is only very recently that both the saddle and inverted conformations have been simultaneously observed within an STM study.^20^

Herein, we present an experimental study of Br_2_TPP (structure in the inset of Figure 1b) on Cu(111) that is found in both the saddle and inverted conformations, depending on the substrate temperature, as observed by STM measurements. Corresponding high-resolution XPS measurements indicate a decrease in the N 1s binding energy shift between the aminic and inverted iminic nitrogen species compared to the aminic and iminic species in the saddle conformations, confirming the predictions of Lepper et al.^13^

STM measurements were performed on a low-temperature ultrahigh-vacuum scanning probe microscope (POLAR, Scienta Omicron GmbH) at the University of Nottingham. Cu(111) surfaces were prepared via cycles of sputtering with Ar ions and annealing. Br_2_TPP molecules were thermally sublimed onto a cooled, clean Cu(111) crystal. STM images were acquired at 4.7 K. All XPS measurements were performed on the Surface and Materials Science endstation at FlexPES beamline at MAX IV Laboratory (Lund, Sweden),^21^ and XPS peak fitting was performed within CasaXPS.^22^

Following deposition of Br_2_TPP molecules onto Cu(111), we observe two different contrasts of the porphyrin molecule within the acquired STM topographs, shown in Figure 1b: type 1 (panels b and d of Figure 1, highlighted in green) and type 2 (panels b and e of Figure 1, highlighted in pink). It can be noted that these two contrasts appear throughout the entire image and within the same scan lines, indicating that the contrast is not an imaging artifact due to tip changes or instabilities. This result indicates that Br_2_TPP on Cu(111) exhibits two distinct electronic structures which, as discussed below, we assign to two different conformations.

Both experimentally observed STM contrasts bear a striking resemblance to DFT-simulated images originally presented by Lepper et al. (see panels d and e of Figure 1). Specifically, type 2 resembles the appearance predicted for the inverted structure, whereas type 1 resembles the prediction of the saddle-shape structure. Within the inverted (type 2) conformation, two bright central lobes are well-defined in both the DFT and experimental STM images. We therefore conclude that, for the as-deposited molecular system, both saddle and inverted conformations are present.

Upon heating this preparation to 303 K, we observe that only one contrast is now visible in the STM images, specifically that related to the inverted (type 2) conformation; this can be seen in Figure 1c. We consider it unlikely that the saddle-shaped porphyrins are desorbing from the surface due to the moderate temperatures used in annealing and because we do not observe any significant change in coverage (multilayers of porphyrins typically desorb at temperatures of over 500 K, with monolayer desorption therefore expected to occur at temperatures significantly higher than those employed here).^23–26^ Instead, the saddle shape seems to be stable on the surface at low temperatures and undergoes a transition to the inverted conformation upon annealing (additional STM images shown in Figure S4). A similar behavior has previously been observed for free-base porphyrins with a range of pendant moieties,^27–30^ indicating that the transition between the two confirmations is applicable to multiple porphyrin variants. In addition, following annealing to this elevated temperature, the debromination and formation of metal–organic frameworks are observed [ubiquitous for Ullmann-type reactions on Cu(111)^31^]. Characterization of the C–Br environment within Br_2_TPP by analysis of the XPS Br 3d region indicates that full debromination occurs following annealing to 0 °C (Figure S9) and results in the formation of short metal–organic TPP oligomers, with the dissociated Br species being adsorbed at the Cu surface (Figure 1c and Figures S4 and S9).

Using temperature-programmed XPS (TP-XPS) measurements, we were able to further investigate the chemical changes occurring within this system during the annealing process. In Figure 2a, the N 1s TP-XPS map, acquired over a temperature range of 90–570 K, is shown. The lower region of the heat map, from 90 to ∼400 K, shows two distinct peaks assigned to nitrogen environments within the porphyrin molecule, aminic (N–H) and iminic (N=), which reduce to a single peak of higher intensity at temperatures above ∼400 K. The presence of these two peaks in the N 1s XP spectra for a free-base porphyrin is well-established.^32–34^ The single peak at elevated temperatures is due to self-metalation of the molecule via the incorporation of a Cu adatom (leading to a N–Cu binding environment) and is equally well-established in the literature.^32–34^ Despite equal numbers of iminic and aminic nitrogen atoms within the molecule, a discrepancy in the intensity ratio of these species exists, i.e., a non-1:1 ratio. This effect has been previously observed and is due to diffractive effects on the photoelectron intensity.^10,35^

**Figure 2:**
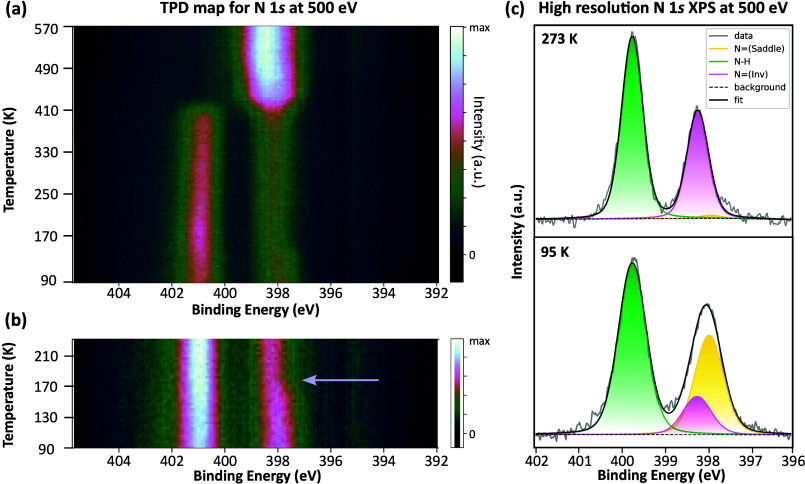
(a) TP-XPS “map” of the N 1s core level for Br_2_TPP on Cu(111) over a temperature range of 95–573 K. Spectra were acquired at photon energy = 500 eV and pass energy = 200 eV. (b) TP-XPS map showing details of the temperature range of 95–233 K. (c) High-resolution N 1s XP spectra taken at 95 K (bottom) and 273 K (top). Spectra were taken at photon energy = 500 eV and pass energy = 100 eV.

An important additional feature and focus of this publication is a small shift in the appearance of the iminic nitrogen peak, observed at 173 K on the TP-XPS map. This can be seen in the expanded section of the TP-XPS map in Figure 2b (indicated by an arrow). In this region, we observe a binding energy shift of 0.27 eV in the iminic peak maxima, from 397.93 to 398.20 eV, showing a clear reduction in peak width and a shift in the peak maxima to a higher binding energy.

Further investigation into the shifting of the iminic nitrogen peak was performed by peak fitting high-resolution XP spectra, shown in Figure 2c, acquired at 95 and 273 K. The data were fitted with three unique species: iminic (N=, 397.93 eV), iminic-inverted [N=(Inv), 398.22 eV], and aminic (N–H, 399.74 eV). Each species were fitted to have a single well-defined binding energy (summarized in Table S1 and Figure S8). These parameters were used to fit the heat map data by only allowing the area of each species to vary as a function of the sample temperature.

Exemplar fitting of three XP spectra (each corresponding to a single line within the TP-XPS data set) acquired before, after, and during the transition from the saddle to inverted conformation can be seen in Figure 3a. The variation in peak area, presented here as a percentage of the total N 1s signal, as a function of the substrate temperature is shown in Figure 3b. These data show that, at the lowest temperatures, an approximately 2:1 ratio between the N= and N=(Inv) species is present. At temperatures between 95 and 163 K, the abundance of N= (yellow line) slowly decreases until, at 163 K, a rapid decrease is observed. A corresponding increase in the N=(Inv) (pink line) species occurs between 95 and 173 K. The total area of the iminic peaks [N= and N=(Inv)] and the aminic peak does not vary significantly over this range, excluding potential molecular desorption (see Figure S5). Indeed, the minor variation in the relative area of the aminic (N–H) peak in this region may be attributed to subtle variations in the position of the aminic N atoms and the resulting changes in the diffractive effects on the photoelectron intensity.

**Figure 3:**
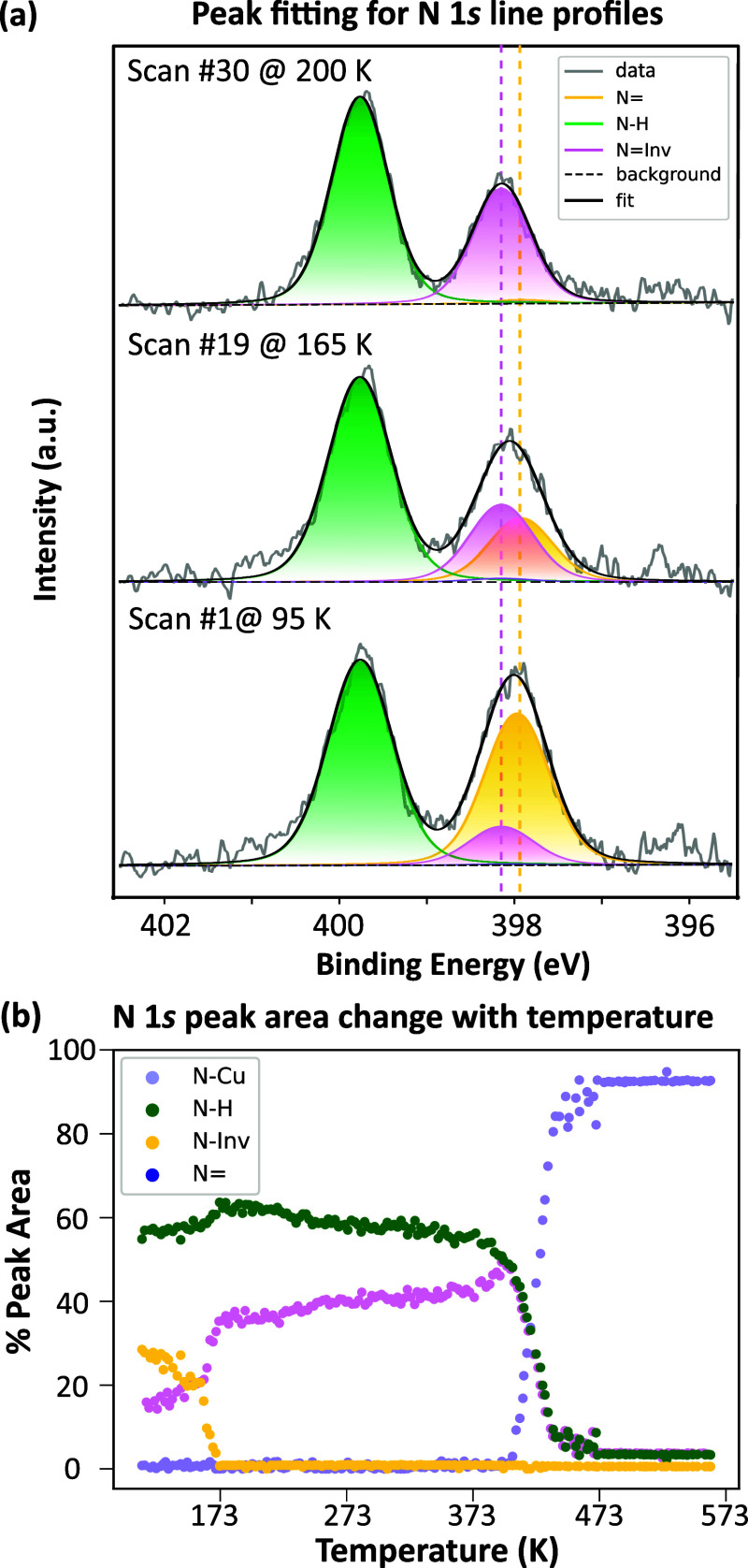
(a) N 1s XPS demonstrating peak fitting of individual spectra, each corresponding to a line from the TP-XPS heat map shown in Figure 2b. Spectra were acquired at photon energy = 500 eV and pass energy = 200 eV. (b) Graph showing peak area change, as a function of the sample temperature, for the chemical species identified within the TP-XPS data.

The decrease in the difference in binding energy between N–H and N=/N=(Inv) is comparable to although smaller than that predicted by Lepper et al. In these experimental measurements, the iminic–aminic splitting for Br_2_TPP was found to be 1.81 eV for the saddle conformation and 1.52 eV for the inverted conformation. Lepper et al. predicted, for 2H-TPP, a splitting of 1.9–2.2 eV for the saddle conformation and 1.4 eV for the inverted conformation. Although there is a clear discrepancy in the magnitude of the decrease in binding energy splitting, the absolute splitting for each species is surprisingly accurate considering the disparate nature of these systems (Br_2_TPP as compared to non-halogenated 2H-TPP). As bromine has significant electronegative character, the electron density across the molecule may be altered, affecting the interaction with the surface. This is likely to influence the absolute binding energies of the nitrogen species, resulting in the discrepancy observed here between (experimentally characterized) brominated and (DFT-calculated) non-brominated molecules.

Previous studies on 2H-TPP have noted the difference in the aminic–iminic binding energy splitting with regards to the substrate material: the splitting is significantly lower on Cu(111), 1.5 eV, compared to Ag(111) and Au(111), 2.0 eV, which has been attributed to a strong molecule–substrate interaction.^36^ The results presented here indicate that this stronger interaction is due to adoption of the inverted conformation on Cu(111). Within Table 1, we present the experimental results from this work alongside literature values for DFT-calculated and experimentally determined values for the aminic–iminic binding energy splitting.

**Table 1:** Comparison of High-Resolution N 1s Splitting Values to DFT Values and Other Previous Works^13,36^

			BE splitting (eV)	
molecule	substrate	method	saddle	inverted
Br_2_TPP	Cu(111)	XPS^a^	1.81	1.52
2H-TPP	Cu(111)	DFT^13^	1.9–2.2	1.40
2H-TPP	Cu(111)	XPS^36^		1.5
2H-TPP	Ag(111)	XPS^36^	2.0	
2H-TPP	Au(111)	XPS^36^	2.0	

a This work.

In summary, we demonstrate via STM characterization that both the inverted and saddle-shaped conformations are present for Br_2_TPP on Cu(111), in contrast to 2H-TPP on the same surface where only the inverted structure has been observed.^13–15^ Our TP-XPS results support the conclusions of the theoretical work by Lepper et al., which shows that saddle-shaped and inverted models of TPP have a clear spectral fingerprint in the N 1s spectra. The binding energy shift between the aminic and iminic N atoms in the saddle-shaped confirmation is measurably larger (1.81 eV) than that of the inverted confirmation (1.52 eV). Our approach provides a route to distinguish between inverted and saddle conformations for porphyrins on other surfaces and crystal facets.

## Methods

STM imaging was performed by using a Scienta Omicron POLAR low-temperature STM system, operating under ultra-high vacuum (UHV) conditions. The system has a base pressure of sub-3 × 10^–10^ mbar. The STM stage was cooled with liquid helium to achieve a temperature of 4.7 K at the sample surface. All STM images were measured in constant current mode with electrochemically etched tungsten tips that may be coated with copper during tip optimization. This was done by controlled indentation into the Cu(111) single-crystal surface. The Cu(111) single-crystal surface was prepared by cycles of Ar ion sputtering for 30 min at 1.0 keV, followed by annealing at 770 K for 30 min. Annealing sample temperatures were measured by thermocouple measurements close to the PBN heater stage. The molecules were deposited (using a Kentax UHV evaporator heated to 250 °C) onto the cleaned surfaces. The Cu(111) sample was removed from the STM stage (at 4.7 K), and transferred to a separate preparation chamber for deposition. Once this was complete, Cu(111) was inserted back into the STM stage with a recorded sample temperature of 110–140 K depending on the speed of transfer and deposition. This indicates that the sample was between 5 and 140 K upon deposition.

The experimental station at FlexPES^21^ features a Scienta SES-2002 hemispherical analyzer (Scienta Omicron) positioned 40° from the incident photon beam, which was used for the photoelectron spectroscopy (PES) measurements. PES measurements were performed at normal emission. The high-resolution XPS spectra were collected with the Cu(111) surface held at 93 K, and the data were collected with a photon energy of 500 eV and a pass energy of 100 eV, in swept mode. TP-XPS was collected at a ramp rate of 2 K/min with a photon energy of 500 eV and a pass energy of 50 eV, in fixed mode. The Cu(111) single-crystal surface was prepared by cycles of Ar ion sputtering for 20 min at 1.0 keV, followed by annealing at 870 K for 10 min. Sample temperatures were measured via a thermocouple placed on the sample plate behind the single crystal. The molecules were deposited (using a Kentax UHV evaporator heated to 250 °C) onto the cleaned surfaces, held at 90 K. To mitigate any potential beam damage during long acquisition times, a defocused beam was used throughout the experiment.

## Supplementary Material



## Data Availability

Data for this article, including STM files and XPS data files are available at The University of Nottingham Research Data Management Repository at 10.17639/nott.7461.

## Notes

Details of TPP conformation, additional STM images, and additional XPS data and fitting details (PDF)
