# Supplementary material for: Experimental Demonstration of a Spectral Fingerprint for the Saddle and Inverted Conformations of Porphyrins on Copper
Source: J Phys Chem Lett. 2025 May 12;16(20):5012–7. doi: 10.1021/acs.jpclett.5c00498 (PMC12105022; doi:10.1021/acs.jpclett.5c00498)
Supplement: Supplementary file 1 [file jz5c00498_si_001.pdf]

Experimental Demonstration of a Spectral Fingerprint for the Saddle and Inverted Conformations of Porphyrins on Copper

Eleanor S. Frampton[a,*], *Ailish Gray*[b], *Michael Clarke*[b], *Matthew Edmondson*[b], *Jonathan Bradford*[b], *David A. Duncan*[c,d] and *Alex Saywell*[b,*]

[a]MAX IV Laboratory, Lund University, 22100 Lund, Sweden

[b]School of Physics & Astronomy, The University of Nottingham, Nottingham, NG7 2RD, UK.

[c]School of Chemistry, University of Nottingham, Nottingham, NG7 2RD, UK.

[d]Diamond Light Source, Harwell Science and Innovation Campus, Fermi Ave, Didcot OX11 0DE, UK.

*Corresponding authors E.F (Eleanor.frampton@maxiv.lu.se) & A.S (alex.saywell@nottingham.ac.uk)

Contents

Tetraphenyl Porphyrin and its Derivatives 2
Additional STM Images 3
High Resolution XPS 4
XPS Fitting Details 6
High Resolution XPS at 95 K and 273 K 7
References 8

Tetraphenyl Porphyrin and its Derivatives

Tetraphenyl porphyrin (2H-TPP, Figure 1 *left*) may take substitutions of different functional groups on the pendant phenyl rings, such as the bromine atoms in this study (see Figure 1b). 2H-TPP displays conformational flexibility *via* rotation of the pendant phenyl rings, as well as within the macrocyclic core. The pyrrolic rings can rotate such that the nitrogen atom is positioned out of plane with the rest of the macrocycle (Figure 1 *right*).

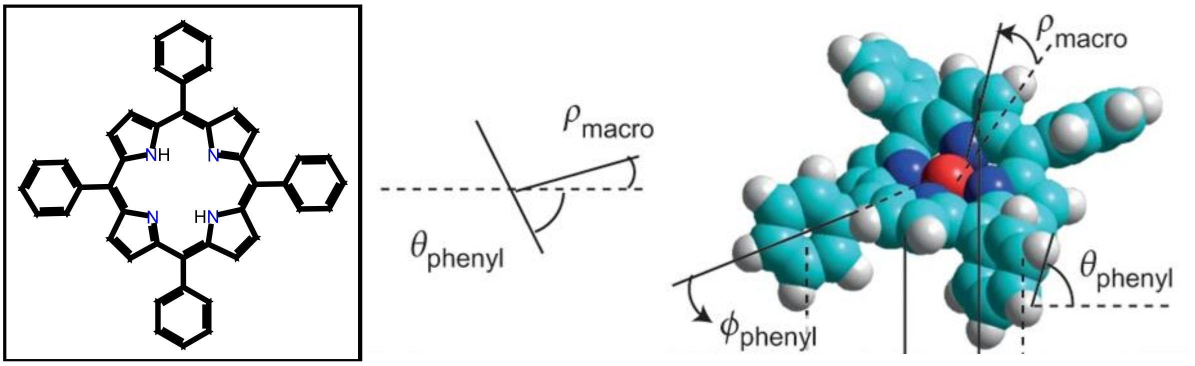

Figure S1 – *Left,* Structure of tetraphenyl porphyrin (as used in the original DFT simulations by Lepper *et. al.*[1]). *Right,* Conformational flexibility of tetraphenyl porphyrin showing possible rotations of the pendant phenyl rings (angle θ) and pyrrole rings within the macrocycle (angle ϕ). Reproduced from (W. Auwärter *et al.*, 2010) refence 2 with permission from the Royal Society of Chemistry.

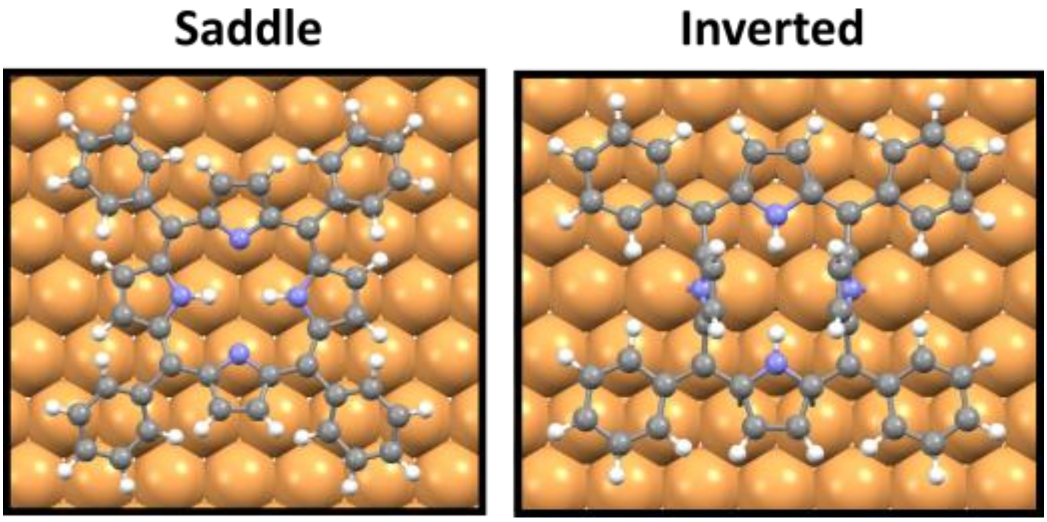

Figure S2 – Top down view of saddle and inverted conformations on the Cu(111) surface. Reproduced with permission from reference 1.

Additional STM Images

Within this work a mixture of the brominated porphyrin species (Br_xTPP) is studied. Each porphyrin is functionalised with between zero and four bromine atoms (*x*=0,1,2,3,4). The sample is referred to as Br_2TPP as this is the majority component. The six potential species (including *cis* and *trans* conformers of the *x*=2 variant) are displayed below in Figure S3. Each variant will present with a different appearance within the STM images (e.g. no. of bright features around the periphery of the molecules) and for each variant the contrast associated with the saddle of inverted conformation (as discussed within the main manuscript) may be present.

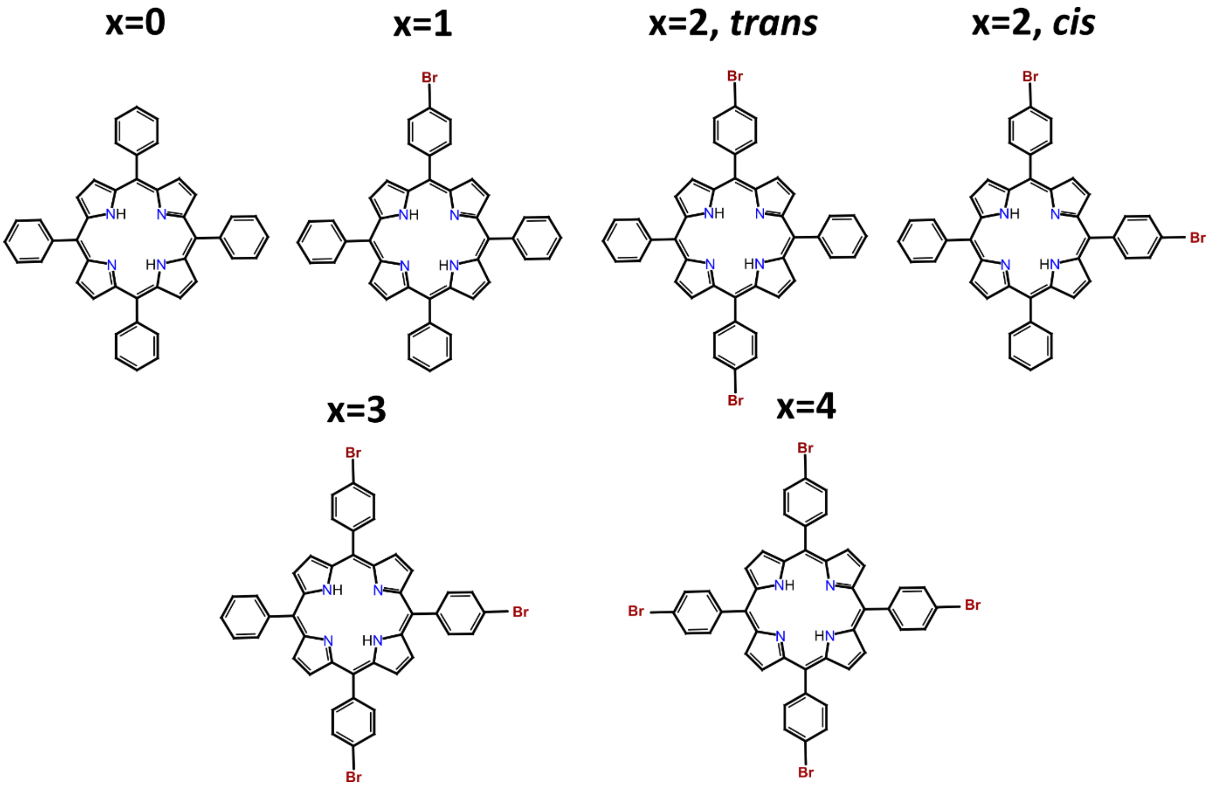

Figure S3 - Variants of Br_xTPP potentially present following deposition onto Cu(111), with *x*=0-4. Note: the skeletal representation of the molecule used illustrates the different potential locations of the bromine atoms and does not represent a specific conformation of the porphyrin core (i.e. saddle or inverted).

Overview images of Br_2TPP on Cu(111) in the as-deposited state and following annealing to 303 K (Figure S4). We observe a distribution of conformations within the as deposited state of: 75% saddle conformation (type 1) and 25% inverted conformation (type 2). Following annealing only the inverted conformation is observed.

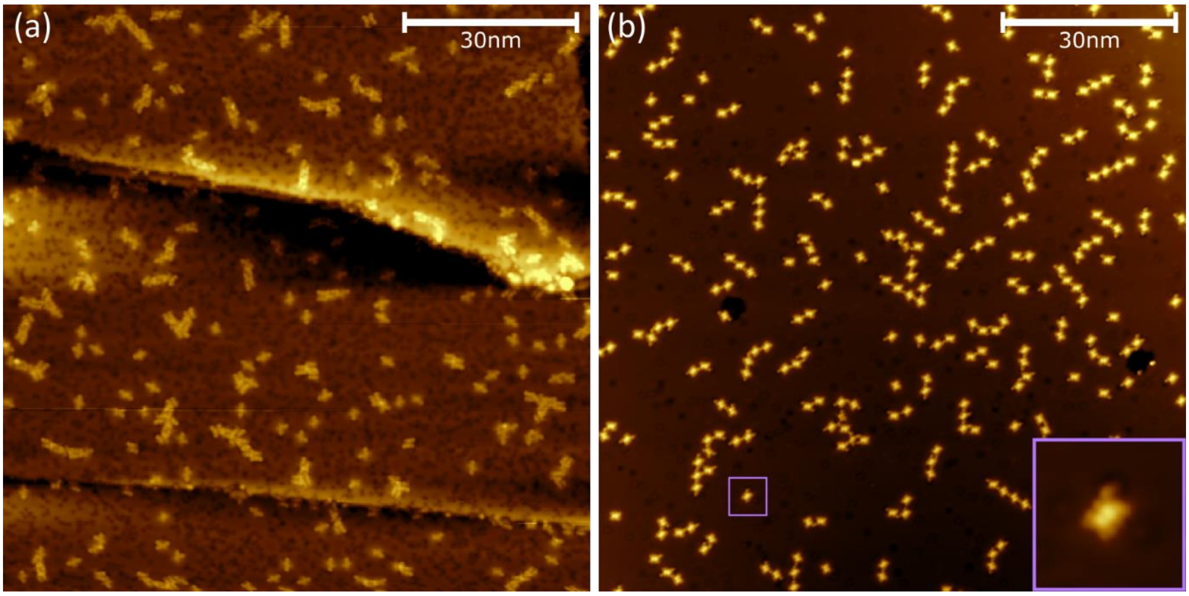

Figure S4 - (a) STM image of as-deposited Br_2TPP on Cu(111). $V_{sample\text{-}bias}$ = -1.8 V, $I_{set\text{-}point}$ = 50 pA. Dimensions: 100 nm x 100 nm. (b) STM image take after heating to 303 K with inset of close-up individual Br_2TPP molecule. $V_{sample\text{-}bias}$ = -1.6 V, $I_{set\text{-}point}$ = 346 pA. Dimensions: 100 nm x 100 nm.

When initially deposited onto the Cu(111) surface we observe individual porphyrin molecules and small clusters (commonly 2-5 molecules per cluster). In Figure S5 some observed structures are highlighted and proposed cluster structures are presented.

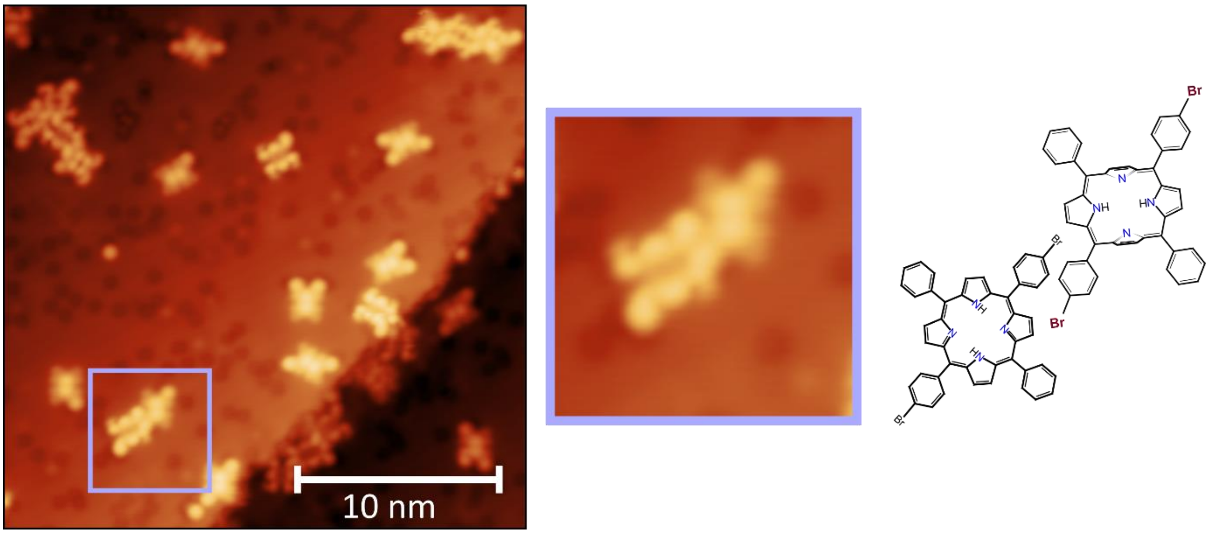

Figure S5 – STM image of as-deposited Br_2TPP on Cu(111). Left: reproduced from Fig. 1b within the main manuscript. Right: Examples of a molecular from Figure 1b and the proposed composition.

High Resolution XPS

The high resolution XPS for the metalated state, taken at 570 K is shown in Figure S4. This spectrum represents all nitrogen atoms bonding to a copper atom taken into the cavity of the molecule from the surface. All nitrogen atoms are in the same environment (bonding to copper) hence only one peak.

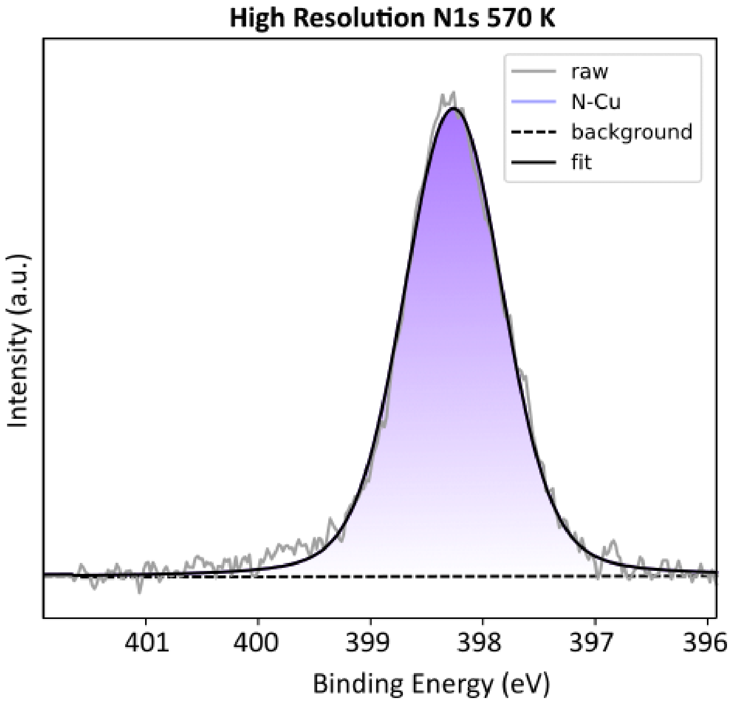

Figure S6- High resolution N 1s XPS spectrum taken at 570 K. Spectrum taken at Photon energy = 500 eV and Pass Energy = 100 eV in swept mode.

XPS Fitting Details

Here the fitting parameters including binding energies and FWHM for the high resolution XPS can be found in the table below. Errors were calculated within the fitting program used (CasaXPS) using Monte Carlo methods. Further details can be found in the program documentation.[4]

Table S1 - Fitting parameters, including binding energies and full-width at half-maximum (FWHM) values, for the high-resolution XPS presented within Figures 2 and 3 of the main manuscript.

	Iminic Nitrogen (N=)			Inverted Nitrogen (N-Inv)			Aminic Nitrogen (N-H)		
	BE (eV)	FWHM (eV)	Area	BE (eV)	FWHM (eV)	Area	BE (eV)	FWHM (eV)	Area
95 K	397.91±0.05	0.8±0.05	0.3±0.2	398.22±0.04	0.79±0.05	0.2±0.2	399.74±0.04	0.9±0.05	0.6±0.2
273 K	397.91±0.05	0.66±0.05	0.01±0.03	398.22±0.05	0.66±0.05	0.3±0.2	399.74±0.05	0.61±0.05	0.5±0.1

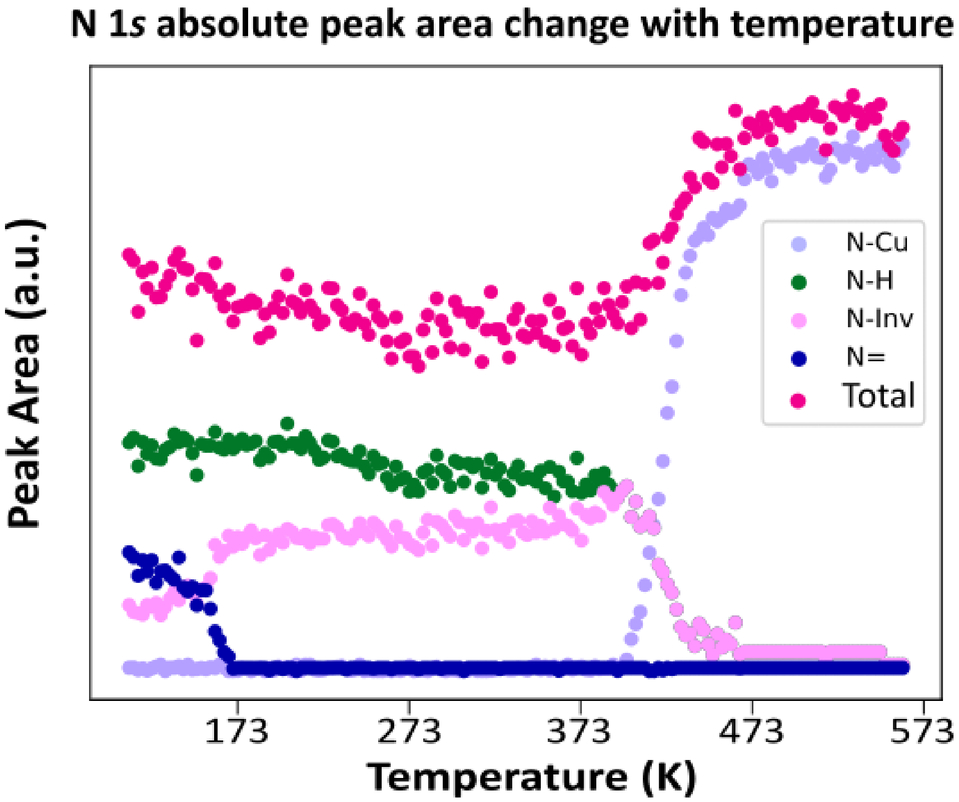

Figure S7 - Graph showing peak area change over time for fitting propagated across TPD map. This shows each component peak as well as the total area.

The total peak area remains relatively constant up to approximately 400 K. This shows that it is unlikely the saddle species has desorbed, and rather transitioned to the inverted species. The increase in total area following 400 K is likely due to the diffractive effects mentioned previously in the main text.

To highlight the difference between the spectra taken at 95K and 273K we have included a differential measurement between the raw data 95 K and the 273 K high resolution XPS (95K -273 K). The differential measurement displays a clear increase in intensity peak in the location of the saddle conformation, indicating that we have at 95 K a clear additional species at this binding energy which we do not have at 273 K. We also observe a significant reduction in intensity at this energy when comparing directly the spectra at 95 K and 273 K.

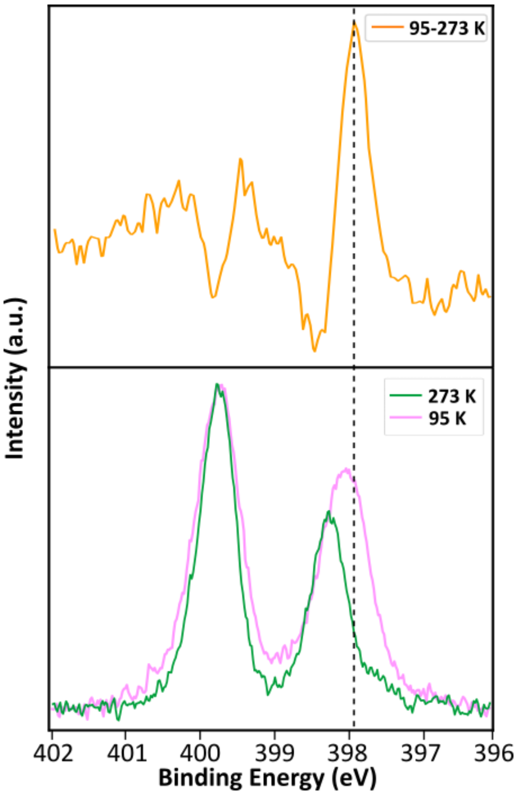

Figure S8 - Top: Differential between high resolution N1 *s* XPS measurements taken at 95 K and 273 K. (95 K - 273 K). Bottom: High resolution XP spectra taken at 95 K (pink) and 273 K (green) overlayed

High Resolution XPS at 95 K and 273 K

High resolution XPS measurements were acquired for the as deposited Br_2TPP species on Cu(111) at 95 K and following annealing to 273 K (Figure S9). XPS measurements show debromination of the molecule occurs following annealing to 273 K (indicating that debromination occurs below, or at, this temperature) and show that bromine remains on the Cu(111) surface following cleavage of the C-Br bond. These chemical environments (C-Br for the intact molecule, and Cu-Br following debromination) are assigned based upon studies for dehalogenation upon coinage metals reported in the literature.[5,6] For the as-deposited molecule we observe features at 70.35 eV and 71.38 eV binding energy (BE) which are assigned to the C-Br environment (purple arrow in Fig. S9) [NB two peaks are expected for a single chemical environment due to spin-orbit splitting (3d3/2, 3d5/2)]. Following annealing to 273 K the pair of peaks (68.68 eV and 69.71 eV BE) observed are assigned to a Br-Cu species (green arrow in Fig. S9), indicating that the Br atoms do not desorb over the temperature range investigated.

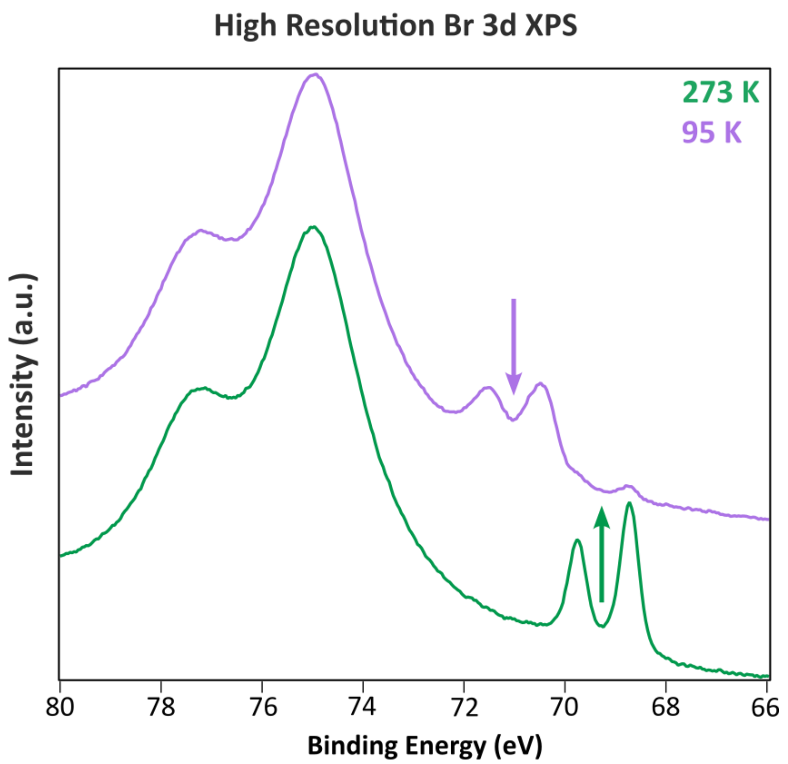

Figure S9 - High resolution Br 3d XPS measurements taken at 95 K and 273 K. Photon Energy = 380 eV, Pass Energy = 50 eV

References

(1) M. Lepper, J. Köbl, T. Schmitt, M. Gurrath, A. de Siervo, M. A. Schneider, H.-P. Steinrück, B. Meyer, H. Marbach and W. Hieringer. "Inverted" porphyrins: a distorted adsorption geometry of free-base porphyrins on Cu (111). *Chem. Commun.*, **2017**, *53*, 8207–8210.

(2) W. Auwärter, K. Seufert, F. Klappenberger, J. Reichert, A. Weber-Bargioni, A. Verdini, D. Cvetko, M. Dell'Angela, L. Floreano, A. Cossaro et al., Site-specific electronic and geometric interface structure of Co-tetraphenyl-porphyrin layers on Ag(111). *Phys. Rev. B*, **2010**, *81*, 245403.

(3) A. Preobrajenski, A. Generalov, G. Öhrwall, M. Tchaplyguine, H. Tarawneh, S. Appelfeller, E. Frampton and N. Walsh. FlexPES: a versatile soft X-ray beamline at MAXIV Laboratory *J. Synchrotron Radiat.*, **2023**, 30, 831–840

(4) N. Fairley, V. Fernandez, M. Richard-Plouet, C. Guillot-Deudon, J. Walton, E. Smith, D. Flahaut, M. Greiner,M. Biesinger, S. Tougaard et al., Systematic and collaborative approach to problem solving using X-ray photoelectron spectroscopy. *Appl. Surf. Sci. Adv.*, **2021**, *5*, 100112

(5) M. Di Giovannantonio, M. El Garah, J. Lipton-Duffin, V. Meunier, L. Cardenas, Y. Fagot Revurat, A. Cossaro, A. Verdini, D. F. Perepichka, F. Rosei, G. Contini. Insight into organometallic intermediate and its evolution to covalent bonding in surface-confined Ullmann polymerization. *ACS Nano* **2013**, *7*, 8190–8198.

(6) Q. Fan, L. Liu, J. Dai, T. Wang, H. Ju, J. Zhao, J. Kuttner, G. Hilt, J. M. Gottfried, J. Zhu. Surface adatom mediated structural transformation in bromoarene monolayers: Precursor phases in surface Ullmann reaction. *ACS Nano* **2018**, *12*, 2267–2274.
